# Clinicopathological Significance of VEGF-C, VEGFR-3 and Cyclooxygenase-2 in Early-Stage Cervical Cancer

**Published:** 2008-03

**Authors:** Xiaoyan Shi, Ling Xi, Danhui Weng, Gang Chen, Xiaohong Song, Peng Wu, Beibei Wang, Juncheng Wei, Shixuan Wang, Jianfeng Zhou, Ding Ma

**Affiliations:** 1 *Cancer biology research center, Tongji Hospital, Tongji Medical College, Huazhong University of Science and Technology, Hubei, P.R. China;*; 2 *Department of Oncology, Renmin Hospital, Yunyan Medical College, 23 Chaoyang road Shiyan, Hubei, P.R. China*

**Keywords:** vascular endothelial growth factor-C, vascular endothelial growth factor receptor-3, cyclooxygenase-2, lymphagiogenesis, lymph node metastasis, cervical cancer

## Abstract

To investigate the roles of VEGF-C, VEGFR-3 and cyclooxygenase-2 (COX-2) in tumor progression and lymph node metastasis. The expression of VEGF-C, VEGFR-3 and COX-2 were examined in 93 cases of surgical speciments of cervical diseases by immunohistochemical staining. The correlation between expression of these factors and tumor aggressiveness was evaluated. The expression levels of VEGF-C and COX-2 were much higher in cervical cancer than in cervical intraepithelial neoplasia (CIN) and in chronic cervicitis. VEGF-C expression correlated with lymph node metastases (*P*<0.01). Multivariate analysis indicated that lymph vessel density (LVD) was associated with the coexpression of VEGF-C and COX-2. Expression of VEGF-C and VEGFR-3 were both in coincidence with lymph node metastasis. VEGF-C and COX-2 may promote the canceration of cervical cancer and that VEGF-C/ VEGFR-3 system had a significant association with the lymphagiogenesis and lymph node metastasis.

## INTRODUCTION

Cervical cancer is the most common cancer in females worldwide, especially in developing country (1). Lymph node metastasis is an important prognostic factor for cervical cancer patients (2, 3). Recently vascular endothelial growth factor-C (VEGF-C), a novel member of the vascular endothelial growth factor family, has been identified as a major regulator of the development of lymphatic vessels (lymphangiogenesis) (4). Previous reports have shown that overexpression of VEGF-C in primary tumors correlates with increased tendency of tumor cell dissemination to regional lymph nodes in a variety of human cancers including breast, prostate, gastric, colorectal, lung and ovarian carcinomas (5-10).

The cognate receptor of VEGF-C, vascular endothelial growth factor receptor -3 (VEGFR-3/flt -4) was known to mainly express in lymphatic endothelium in adult and therefore was regarded as a special marker of lymphatic endothelium. The density and distribution of lymphatic vessels could be detected by VEGFR-3/flt -4 antibody (11-16). It has been investigated that the binding of VEGF-C to its special receptor flt-4 could induce tyrosine phosphorylation of receptors, activate MAPK via intracellular kinase reaction and finally promote proliferation of lymphatic endothelium and lymphangiogenesis (17, 18).

Cyclooxygenase (COX)-2, a pleiotropic enzyme that mediates many physiological functions, may promote carcinogenesis, tumor proliferation and metastasis by mediating pathological processes that affect mitogenesis, cellular adhesion and immune surveillance (19-21). Therefore, overexpression of COX-2 has also been considered an indicator of tumor invasiveness and aggressiveness as well as a predictor of metastatic potential in various types of cancers, including uterine cervical cancer (20-23). A significant positive correlation between COX-2 and VEGF-C expression has been observed in several human tumors (24-26). Furthermore, VEGF-C was demonstrated to be one of the major downstream genes of COX-2 and up-regulated by COX-2 through EP1/Src/HER-2/Neu signaling pathway in human lung adenocarcinoma cells. COX-2-specific inhibitors significantly attenuated the endogenous VEGF-C expression level in tumor cells (27). These Studies suggest that COX-2 may be a regulator of VEGF-C expression in malignancies.

In the present study we investigated the expression of VEGF-C, its receptor VEGFR-3 and cyclooxygenase-2 (COX-2) and their relationship with tumor progression and lymph node metastasis in 93 patients with cervical cancer.

## MATERIALS AND METHODS

### Patients and Tissues

The enrolled specimens in this study were from 93 patients (mean age 41.65 years, range 27-69 years) with informed consent, including 45 cases of invasive cervical carcinoma (5 in Stage I a, 20 in stage I b, 10 in stage II a, 10 in stage II b), 30 cases of cervical intraepithelial neoplasia (CIN) and 18 cases of chronical inflammation undergoing surgical treatment in Tongji Hospital from 2002 to 2006. Diagnosis was established preoperatively by punch biopsy or cone excision and verified by post-operation pathological diagnosis. All tissues were fixed with formalin and paraffin embedding. None of the patients received any radiotherapy or chemotherapy prior to the study. All cervical carcinoma patients were treated with radical hysterectomy and pelvic lymph node dissection. 26 of 45 patients with invasive carcinoma have regional lymph node metastasis.

### Reagents

Polyclonal rabbit anti-VEGF-C (H-190), polyclonal rabbit anti-flt-4 (C-20) was purchased from Santa Cruz Biotechnology (Santa Cruz, Calif.). Rabbit polyclonal antibody specific for human cyclooxygenase-2 (COX-2) was purchased from Cayman Chemical (Ann Arbor, MI, USA).

### Immunohistochemistry

Consecutive 4μm sections of each specimen were immunostained for VEGF-C, VEGFR-3 and COX-2. Immunohistochemical staining was performed by the immunoperoxidase technique. Sections of deparaffinized tissues in xylol were heated for 15 min in a microwave oven in 10 mmol/l citrate buffers (pH 6.0). After cooling for 30 min and washing in PBS, endogenous peroxidase was blocked with 3% H_2_O_2_ for 20 min, followed by incubation with PBS containing 10% normal goat serum for 30 min. Then the primary antibodies were incubated at 4°C overnight (The primary antibody dilution of VEGF-C, VEGFR-3 and COX-2 was 1:200, 1:300 and 1:50, respectively), followed by detection by the SP kit (NEW Life Science Products, Inc., Boston, Mass.) according to the manufacturer’s instructions. Alternatively, the detection was performed using a biotinylated anti-rabbit Ig and avidin combined with biotinylated peroxidase complex. Sections were incubated with avidin D and biotin solutions. The color was developed with diaminobenzidine (DAB) supplemented with hydrogen peroxide and counterstaining was performed using hematoxylin. A tissue block of breast cancer with a high microvessel density served as a positive control. Negative controls were done by PBS instead of the primary antibody.

All slides were investigated by two pathologists who were blinded to the cases to prevent any interindividual classification errors. According to the staining percentage of immunoreactive tumor cells, if the distributions of VEGF-C or COX-2 immunoreactivity no less than 10%, the sample was classified as positively stained. If no cell stained or the staining percentage less than 10%, the sample was classified as negatively stained. If the percentage of VEGF-C immunoreactivity was more than 50%, the slide was defined as a strong positive (+++). Accordingly percentage of VEGF-C immunoreactivity between 30% and 50% was defined as moderate positive (++) and 10%-30% weak positive (+) respectively. VEGFR-3 immunoreactivity in lymphatic endothelium or tumor cells more than 10% was defined as a positive result. Lymph vessel density (LVD) was determined by counting the numbers of VEGFR-3 positive vessels in 5 hot areas and getting the mean value under a low power light microscope (100×). Lymphovascular space involvement in immunostained slides was considered positive if at least one tumor cell cluster was clearly visible in a decorated lymphovascular space (20, 28).

### Statistical analyses

The expression of VEGF-C, VEGFR-3 and COX-2 among different cervical diseases was analyzed using Kruskal-Wallis Test. Correlation among VEGF-C, VEGFR-3 and COX-2 expression was analyzed using the Pearson’s correlation. These analyses were performed utilizing the SPSS 11.0 software. For all tests, *P*≤0.05 was considered statistically.

## RESULTS

### Expression of VEGF-C, VEGFR-3 and Cox2 in cervical tissues

**Figure 1:**
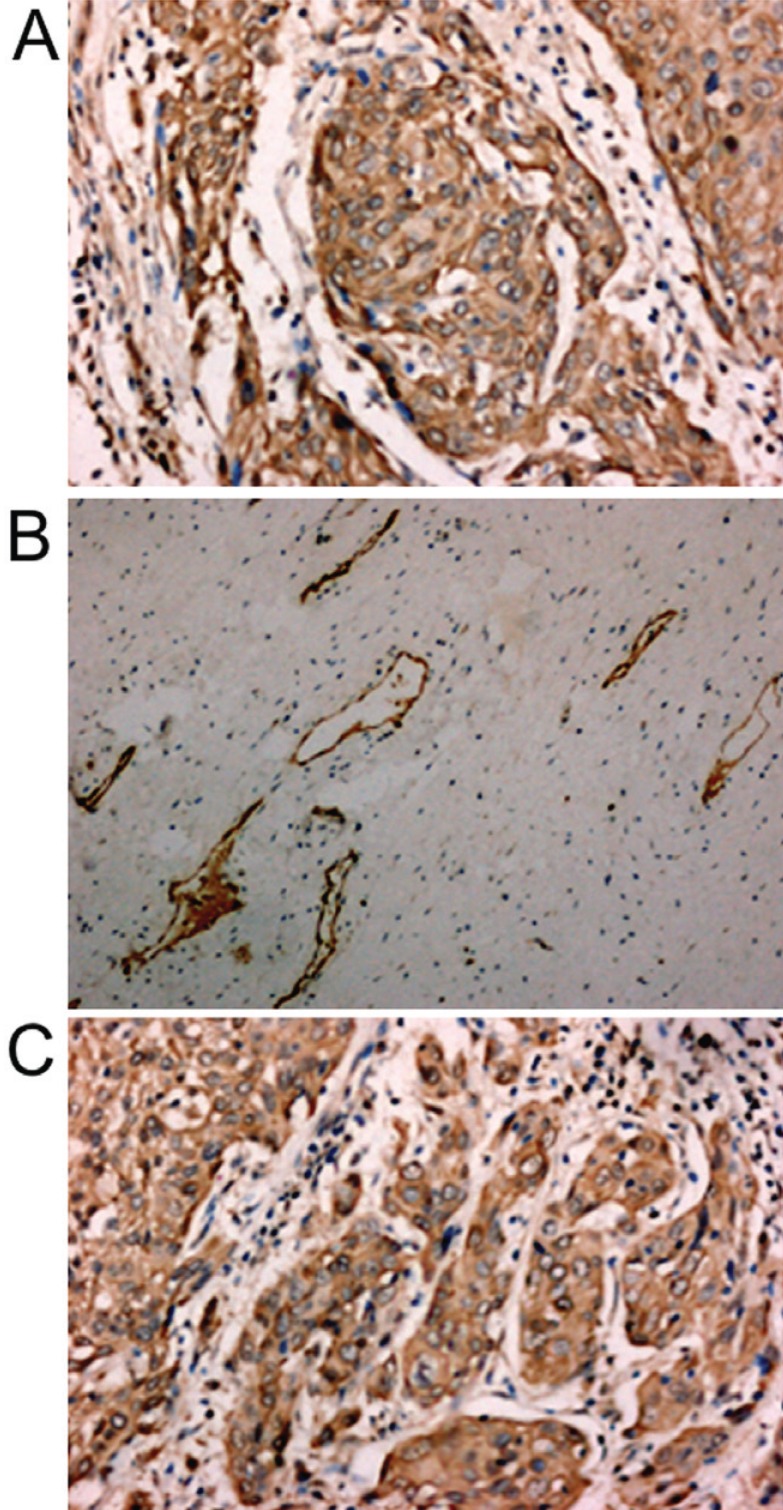
Immunohistochemical staining of VEGF-C, VEGFR-3 and COX-2. A, VEGF-C was stained in the cytoplasm of cervical cancer cells. B, VEGFR-3 expressed in the lymphatic vessels of the stromal adjacent to tumor nest. C, COX-2 was stained in the cytoplasm of cervical cancer cells.

The immunohistochemical expression of VEGF-C and Cox2 was observed in the cytoplasm of tumor cells. Of the 93 patients, 9 cases (50%) were positive for VEGF-C in chronic cervicitis, 23 cases (76.6%) positive in cervical intraepithelial neoplasia (CIN) and 40 cases (88.9%) positive in cervical cancer (Table 1). In the chronic cervicitis tissue, moderate VEGF-C immunoreactivity was present in the cytoplasm of stroma cells and a weak immunoreactivity in some smooth muscle cells of blood vessels (data not shown). In the CIN and cervical cancer tissue including 26 patients with lymph node metastases, moderate to strong VEGF-C immunoreactivity was present in the cytoplasm of many cancer cells (Figure 1A).

**Table 1:** The expression of VEGF-C and COX-2 in cervical diseases

Cervical disease	Case	VEGF-C				COX-2	
		-	+	+ +	+ + +	-	+

**Inflammation**	18	9	6	3	0	18	0
**CIN**	30	7	11	9	3	22	8
**Invasive cancer**	45	5	9	17	14	28	17

Kruskal-Wallis Test showed that the expression level of VEGF-C and COX-2 between different cervical diseases was significantly different (*P* value was 0.0001 and 0.008 respectively). Bivariate showed that expression of VEGF-C correlated with that of COX-2 (*p*=0.009).

Of the 45 cervical cancer patients studied, 17 cases (37.8%) were positive for COX-2 expression in cytoplasm (Figure 1C). 8 cases (26.7%) of 30 CIN tissues expressed COX-2 while none of chronic cervicitis expressed COX-2 (*P*<0.01) (Table 1). Furthermore the immunoreactivity of COX-2 was associated with that of VEGF-C in the specimens (*P*<0.01) (Table 1).

Strong immunoreactivity of VEGFR-3 was detected in the lymphatic endothelium in the stroma adjacent to the tumor nest (Figure 1B). In addition, moderate immunoreactivity of VEGFR-3 was also observed in the cytoplasm and membrane of cervical cancer cells. LVD was significantly different between different cervical diseases (Table 2).

**Table 2:** The expression of VEGFR-3 in cervical diseases

Cervical disease	N	X ± *s*

**Inflammation**	18	2.45 ± 0.60
**CIN**	30	3.84 ± 0.60
**invasive cancer**	45	5.84 ± 1.00

ANOVA Test showed that the expression level of VEGFR-3 between different cervical diseases was significantly different (F =37.17 and *p*=0.0001 respectively). Kruskal-Wallis Test showed that the expression level of VEGFR-3 was associated with that of VEGF-C (*p*=0.00003).

### Correlation between expression of VEGF-C, VEGFR-3, COX-2 and LVD and Lymphnode metastasis

The association between expression of VEGF-C, COX-2, VEGFR-3 and LVD was demonstrated by serial sections in cancer cells. There was a positive relationship between the expression of VEGF-C and VEGFR-3 (*P*<0.01). The number of VEGFR-3 positive vessels increased with the expression of VEGF-C and COX-2. Meanwhile there were few VEGFR-3 positive vessels in VEGF-C and COX-2 negative tissues. Multivariate analysis indicated that lymph vessel density (LVD) was associated with the coexpression of VEGF-C and COX-2. Expression of VEGF-C and VEGFR-3 was both in coincidence with lymph node metastasis. However, expression of COX-2 was not associated with lymph node metastasis (Table 3).

**Table 3:** Relationship between lymph node metastasis and VEGF-C and COX-2 in invasive cervical carcinoma

Lymphnode metastasis	N	VEGF-C				COX-2	
		-	+	+ +	+ + +	-	+

+	26	5	6	10	5	16	10
-	19	0	3	7	9	12	7

Bivariate showed that expression of VEGF-C correlated with lymphnode metastasis (*p*=0.005) while COX-2 did not correlate with lymphnode metastasis (*p*=0.830).

## DISCUSSION

Many cancers metastasize to regional lymph nodes, and a positive nodal status often correlates with a poor prognosis of patients. However, the mechanisms of lymphatic metastasis have not been investigated in detail. Recent studies have demonstrated that the expression of VEGF-C is enhanced in various solid tumors, suggesting the possible contribution of VEGF-C to nodal metastasis, possibly through lymphangiogenesis (28, 29). Number of clinical studies has shown a positive correlation between VEGF-C expression and risk of lymph node metastasis in various cancers. Moreover, the increase in VEGF-C level from primary tumor to metastatic lymph node might be a prognostic indicator (30).

In the present study, our results showed that VEGF-C expressed principally in the cytoplasm of tumor cells which was consistent with the reports (31-33). In addition, a small amount of VEGF-C was detected in the stroma cells and the smooth muscle cells of blood vessels adjacent to the tumor nest (data not shown). We also observed that the expression level of VEGF-C increased with the progression of cervical intraepithelial neoplasia (CIN) to cervical invasive carcinoma, which suggested that VEGF-C might promote the canceration of cervical cancer.

Study suggested that cancer cells immerged into lymphatic vessels in early stage and metastases to their draining lymphonode was frequently found in human tissues. Morphologically, the dilated lymphatic vessels with cancer cells immerging often located at the periphery of malignant tissues (34). In the present study the expression of VEGFR-3 was not only detected at the lymphatic endothelium but also at the tumor cells and the capillaries of the stroma adjacent to the tumor nest. The serial sections showed that the expression of VEGFR-3 was detected in VEGF-C positive cancer cells. Meanwhile VEGFR-3 positive vessels were rarely found in the VEGF-C negative tissues. Interestingly, we also observed that there were many expanded lymphatic vessels with several cancer cells inside the areas where VEGF-C and VEGFR-3 strongly expressed. However, we identified functional lymphatic vessels within peritumoral stroma, instead of within tumors, together with previous studies (35-37). A reasonable explanation might be that neoplastic cells grown in a confined space generate mechanic stress which may compress or inhibit the development of lymphatic channels inside the tumor (35).

Multivariate analysis indicated that the expression of VEGF-C and VEGFR-3 correlated with lymph vessel density (LVD). High peritumoral lymphatic microvessel density (LMVD) and lymphatic infiltration were tightly associated with lymph node metastases as increased lymphatic windows provided more opportunity for cancer cells to invade and metastasize to lymph nodes (35). It was reported that VEGF-C has effects on the lymphovascular endothelium through its autocrine signal network by enhancing the permeability, promoting the migration and proliferation of endothelial cells and thus facilitating malignant cells to enter into lymphatic vessels (28). Taken together we may conclude that expression of VEGF-C was positively related to expression of VEGFR-3 and the concomitance of the VEGF-C/VEGFR-3 system facilitates the lymphatic proliferation and invasion.

Clinical studies have demonstrated that COX-2 is an independent prognostic indicator in cervical cancer patients (38, 39). Recent reports showed that COX-2 implicated in tumor lymphangiogenesis through an upregulation of VEGF-C expression (11). In our study COX-2 was positively detected in 17 (37.8%) cervical cancer patients, which was consistent with previous reports (28). Statistical analysis manifested that COX-2 expression level was related to that of VEGF-C. Moreover, the coexpression of VEGF-C and COX-2 correlated with lymph vessel density (LVD) of the stroma adjacent to the tumor nest. However, there was no significant correlation between COX-2 immunoreactivity and the expression level of VEGFR-3 as well as lymph node metastasis.

In conclusion, our results indicate that VEGF-C and COX-2 may promote the canceration of cervical cancer and that VEGF-C/ VEGFR-3 system have a significant association with the lymphagiogenesis and lymph node metastasis. Therefore, determination of VEGF-C in biopsy specimens may be useful as a predictor of pelvic lymph node metastasis. COX-2 plays an important role in the lymphatic proliferation and spread through the VEGF-C mediated lymphangiogenic pathway in cervical cancer. VEGF-C/VEGFR-3 could be a potential therapeutic target for cervical cancer.
